# A possible link between loading, inflammation and healing: Immune cell populations during tendon healing in the rat

**DOI:** 10.1038/srep29824

**Published:** 2016-07-13

**Authors:** Parmis Blomgran, Robert Blomgran, Jan Ernerudh, Per Aspenberg

**Affiliations:** 1 Orthopaedics, Department of Clinical and Experimental Medicine, Linköping University, Sweden; 2 Division of Medical Microbiology, Department of Clinical and Experimental Medicine, Linköping University, Sweden; 3 Division of Clinical Immunology, Department of Clinical and Experimental Medicine, Linköping University, Sweden

## Abstract

Loading influences tendon healing, and so does inflammation. We hypothesized that the two are connected. 48 rats underwent Achilles tendon transection. Half of the rats received Botox injections into calf muscles to reduce mechanical loading. Cells from the regenerating tissue were analyzed by flow cytometry. In the loaded group, the regenerating tissue contained 83% leukocytes (CD45^+^) day 1, and 23% day 10. The M1/M2 macrophage ratio (CCR7/CD206) peaked at day 3, while T helper (CD3^+^CD4^+^) and T_reg_ cells (CD25^+^ Foxp3^+^) increased over time. With Botox, markers associated with down-regulation of inflammation were more common day 5 (CD163, CD206, CD25, Foxp3), and M1 or M2 macrophages and T_reg_ cells were virtually absent day 10, while still present with full loading. The primary variable, CCR7/CD206 ratio day 5, was higher with full loading (p = 0.001) and the T_reg_ cell fraction was lower (p < 0.001). Free cage activity loading is known to increase size and strength of the tendon in this model compared to Botox. Loading now appeared to delay the switch to an M2 type of inflammation with more T_reg_ cells. It seems a prolonged M1 phase due to loading might make the tendon regenerate bigger.

Mechanical loading has a profound influence on tendon healing. Reduced loading has been shown in numerous models to impair healing^1^. In this work we use a rat model for Achilles tendon healing, in which unloading by several different methods has been shown to reduce the size and strength of the healing tissue to a large extent, compared to normal full cage activity^2^,^3^,^4^. More than two thirds of the strength can be lost^4^. Recently, we have noticed that anti-inflammatory drugs impair tendon healing mainly by impairing the response to loading or microdamage, and also that the expression of inflammation-related genes was involved^5^,^6^,^7^. This points to a possible connection between loading, inflammation and healing.

In order to shed some light on this possibility, we need to create a reasonably simple, yet comprehensive way of describing the status of inflammation at any given time point. By analyzing the cell populations from healing tissue by flow cytometry with markers for several inflammatory and regulatory cell types, we can describe the composition of the population and use it as an “inflammatory signature”. Changes in the signature over time can then show how the inflammatory reaction is influenced under varying conditions. To our knowledge, no such detailed study of the immune cell population during tendon healing has been published.

Among regulating cell populations, different subsets of macrophages hold a central position in the immediate response to infectious threats as well as to tissue damage^8^. M1 macrophages are induced under inflammatory conditions and exert proinflammatory actions. M2 macrophages constitute a heterogeneous population, in general being involved in healing and performing anti-inflammatory and homeostatic actions. T_reg_ cells are the main regulators in the adaptive response and they perform anti-inflammatory actions that participate in shifting the type of inflammation towards supporting early tissue repair^9^.

The general hypothesis for this study was that inflammation is influenced by mechanical loading. It is thought that a shift from the early M1 type response to a later M2 type is crucial for the initiation of the regenerative phase of healing. We therefore tested the specific hypothesis that loading would influence the ratio between macrophages expressing M1 (CCR7) and M2 (CD206) markers day 5. We chose to use CD206 because these cells (M2a macrophages) are the result of polarization by IL-4 and IL-13 and they are known to stimulate fibroblast proliferation and collagen production via TGF-β and other mechanisms^10^,^11^. After having confirmed that loading influences the ratio between pro and anti-inflammatory macrophages, we formulated a second hypothesis, namely that the T cell population would change in a similar way. This was tested using the ratio between anti-inflammatory T_reg_ cells and T helper cells, comparing the loaded and unloaded groups on day 5 (ratio CD3^+^CD4^+^CD25^+^Foxp3^+^/CD3^+^CD4^+^). Other changes in the inflammatory signature are described by statistical post-hoc testing.

## Results

### Hypothesis tests

The ratio M1/M2 (CCR7/CD206) at day 5 was 1.8 (95% CI 1.4 to 2.1) with Botox, and 2.8 (95% CI 2.4 to 3.2) with full loading (p = 0.001; Table 1) indicating that loading shifted the inflammatory reaction towards a more M1 macrophage dominated pattern. Although not the preselected time point, the difference was even greater day 3 (Table 1).

The ratio T_reg_ cells/T helper cells (CD3^+^CD4^+^CD25^+^Foxp3^+^/CD3^+^CD4^+^) at day 5 was 0.072 (95% CI 0.05 to 0.09) with Botox, and 0.025 (95% CI 0.017 to 0.033) with full loading (p < 0.001; Table 1) indicating that loading reduced the relative number of T_reg_ cells.

### Immune cell signature in loaded tendons during the course of healing

**Figure 1:**
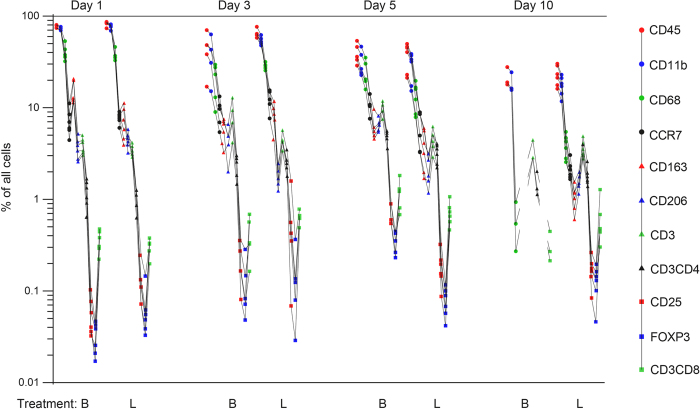
Inflammatory signature of tendon healing day 1, 3, 5 and 10, with and without reduced loading by Botox. Note change in pattern over time. Each line describes cell counts from one rat. The Y-axis presents data on a log scale. All markers are expressed as percent of all cells. Full loading is indicated by L, and Botox by B. The points on the line represents the immune cell markers in the following order: CD45 (leukocytes), CD11b (phagocytes), CD68 (pan-macrophages), CCR7, CD163, CD206 (macrophage subtypes), CD3, CD4, CD25, Foxp3, CD8a (lymphocytes and lymphocyte subtypes). On day 10 several markers were below detection level in the Botox group, hence the interrupted lines. N = 6 for most groups, with exceptions, see text.

The pattern of immune cells (“immune cell signature”) was consistent and similar in all rats (Fig. 1). Initially, the tendon callus contained predominantly leukocytes, i.e. CD45^+^ cells. Their proportion decreased from 83% (95% CI 78.2 to 87.9) of all cells day 1 to 23% (95% CI 16.9 to 29) day 10 (p < 0.001; Fig. 1). Most of these cells were phagocytic (CD11b^+^), but the proportion of T cells (CD3^+^) of all inflammatory (CD45^+^) cells increased from 4% day 1 (95% CI 3.4 to 4.5), to 18% day 10 (95% CI 14.1 to 21.2) (p < 0.001; Supplementary Figure S1).

At all time-points, the majority of CD45^+^ cells were CD11b^+^, i.e. mainly neutrophils, monocytes, macrophages, or dendritic cells. We considered only CD11b^+^ and CD68^+^ double positive cells as macrophages. Using this rather strict criterion, 47% (95% CI 37.4 to 56.5) of the CD45^+^ cells were macrophages at day 1, which then decreased over the course of healing, so that at day 10 only 17% (95% CI 12.6 to 22.1) of the CD45^+^ cells were macrophages (p < 0.001; Supplementary Figure S2). The percentage of CD11b^+^ cells remained constant between day 3 and day 10. The 2.7-fold difference in macrophages between these time-points therefore indicates that another CD11b^+^ cell type than macrophages was recruited to the site of healing.

**Figure 2:**
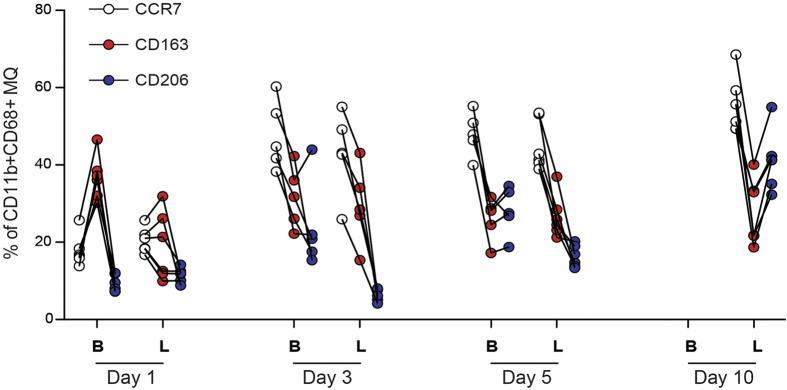
The proportion of macrophage subsets presented as percent of all macrophages (MQ; CD11b^+^CD68^+^) in the healing tissue over time. CCR7 corresponds to M1 macrophages, CD163 and CD206 correspond to different types of M2 macrophages. Full loading indicated by L, Botox by B. Each line describes cell counts from one rat. At day 10 the cell number in the Botox group was too low to show the macrophage subtypes.

The different macrophage subtypes (as percentage of CD11b^+^CD68^+^) showed a consistent pattern. In each rat, CD206^+^ (M2a) macrophages was lower than CD163^+^ (M2c) macrophages at days 1, 3 and 5. In each rat, CD206 was higher than CD163 at day 10 (Fig. 2). CCR7^+^ (M1 macrophages) showed an early abundance and were already high at day 3. The CD163^+^ macrophages, although exhibiting a small increase from day 1 to day 3, were more or less constant over time.

T cells constituted a small fraction of the CD45^+^ cells at day 1 (1.2% CD3^+^CD4^+^, and 0.36% CD3^+^CD8a^+^). Both CD3^+^CD4^+^ and CD3^+^CD8a^+^ T cells increased over time (r = 0.84, p < 0.001 and r = 0.79, p < 0.001 respectively) although CD3^+^CD4^+^ cells were at least 3 times as many as the CD3^+^CD8a^+^ cells at all time-points (Supplementary Figure S3).

**Figure 3:**
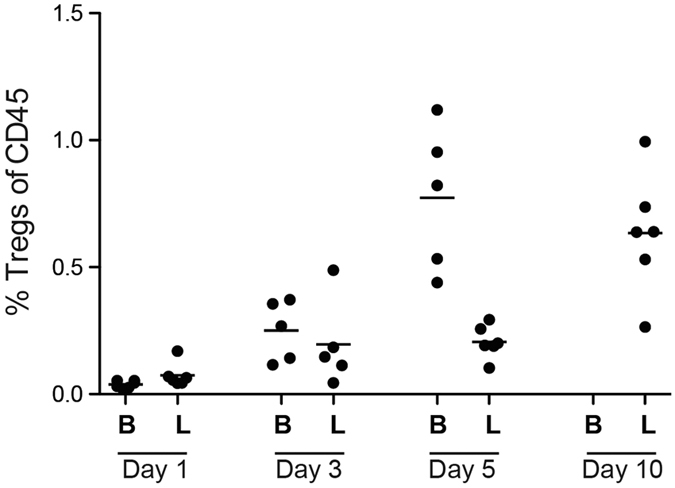
The proportion of T_reg_ cells (CD3^+^CD4^+^CD25^+^Foxp3^+^) presented as percent of all leukocytes (CD45^+^) during healing. Full loading indicated by L, Botox by B. At day 10 the cell number in the Botox group was too low to perform accurate analysis of T_reg_ cells.

The activation marker CD25 suggested that an increased proportion of T cells were activated at day 3. Further including Foxp3 to define T_reg_ cells, they showed a gradual increase with the largest increase taking place between day 5 to day 10 (p = 0.002; Fig. 3). This increase might suggest that the absolute number or T_reg_ cells was maintained at day 10, since the absolute number of CD45^+^ cells on day 10 had dropped to approximately 1/6 of that at the previous time-point.

### Effect of Botox on the immune cell signature

**Figure 4:**
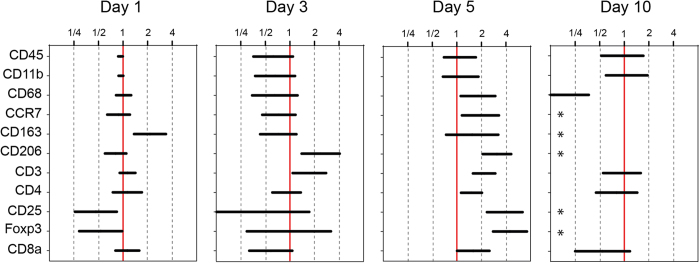
Difference between full loading and Botox in the number of marker positive cells out of all cells. 95% confidence intervals for the differences are shown. Values are expressed as a ratio Botox/full loading. Stars (*) indicate extreme values outside of the graph: at day 10, cells labelled with CCR7, CD163, CD206, CD25, and Foxp3 were too few in the Botox group to perform accurate analysis, while still present with full loading.

Within the Botox treated group there was also a consistent immune cell signature (Fig. 1). The proportion of leukocytes (CD45^+^) of all cells decreased over time in both the Botox and loaded groups with no obvious difference, but there were striking differences in the composition of the immune cell populations (Fig. 4). Macrophages in general (CD11b^+^, CD68^+^) and their subgroups were increased by Botox day 5 (CCR7, p < 0.001; CD163, p = 0.006; CD206, p < 0.001) but then almost disappeared at day 10 (Fig. 4). In contrast, they were present in quantifiable numbers in the full loading group.

A similar pattern was found for T_reg_ cells (CD3^+^CD4^+^CD25^+^Foxp3), which were dramatically increased in the Botox group at day 5, (p = 0.01) but had virtually disappeared at day 10 (Figs 3 and 4). In contrast, they remained quantifiable in the full loading group.

In summary, cells that are thought to down-regulate the inflammation were increased in the Botox group at day 5, and then disappeared.

## Discussion

Unloading with Botox is known to impair healing of rat Achilles tendons^4^. We now found that this impairment is associated with changes in the inflammatory cell signature. Before discussing further, we must consider what unloading with Botox in this rat model means in relation to clinical situations.

Rats are adapted to avoid predation, and therefore may avoid showing vulnerability by limping or slowing down in case of injury. Therefore, the loading of their healing Achilles tendon during free cage activity (full loading) is probably close to maximal, and it has been shown to be associated with microdamage within the healing tissue under the circumstances of this experiment^12^. Indeed, full loading shows effects on biomechanical and gene expression parameters similar to repeated traumatization by needling^4^,^13^. In contrast, human patients normally don’t apply maximal load on their injured tissues, and micro-injury similar to the rats is probably uncommon, except possibly in highly ambitious athletes. Therefore, the rat group with reduced loading due to Botox paralysis probably better mimics the clinical, human situation. We thus regard rats with Botox-induced paralysis as showing the “standard” healing progress, and full loading as a model for increased or exaggerated loading in humans.

With load protection due to Botox, the proportion of cells thought to turn off the inflammatory response were increased day 5 (CD163, CD206, and CD25, Foxp3). At day 10, these cells no longer remained in measurable amounts, meaning that the inflammation had resolved between days 5 and 10. In contrast, full loading prevented the increase in the proportion of down-regulating cells days 3 and 5, and their continued presence indicate that the resolution process was still ongoing day 10. This suggests that loading during free cage activity prolonged the early type of inflammatory reaction, dominated by M1 macrophages, and delayed the switch to a “constructive” type of inflammation with more M2 macrophages and T_reg_ cells. Previous data have shown that loading by unrestricted cage activity is associated with an increased strength of the healing tendon^4^. However, this increase in strength is due to an increased mass of the healing tissue, i.e. increased cross-sectional area, mainly without an improvement in mechanical quality. Still, loading can improve also the mechanical material properties, but this effect is mainly seen in the low loading range, i.e. when loading is increased from minimal to the level of loading that corresponds to the Botox model^4^.

There is no clear picture of the influence of macrophages on tendon or ligament healing. Some studies report that macrophage depletion or deficiencies are associated with improved quality of the healing tissue^14^,^15^,^16^, sometimes together with a decreased mass of tissue^15^. These studies looked at the effect of absence of macrophages during the entire healing process. However, early inflammation definitively has a positive effect, as evidenced by studies with NSAIDs^17^ and experiments where macrophages have been specifically inhibited^18^,^19^. The role of mechanical loading in conjunction with macrophages has been studied in a rat model for tendon to bone healing, where immobilized animals had fewer macrophages (CD68^+^) in total at 2 and 4 weeks, but their proportion of CD163 positive cells at 2 weeks was increased as estimated by immunohistochemistry^20^. These results might concur with our finding that immobilization shortens the time to resolution of inflammation. However, since CD68 may also be expressed on primary fibroblasts and endothelial cells the interpretation of its expression by immunohistochemistry is uncertain^21^. In contrast, flow cytometry analysis utilizing a combination of verifying markers such as CD45 and CD11b together with CD68 will better ensure accurate detection of macrophages.

The fact that CD45^+^ cells constituted a considerable fraction of the cells still at day 10 in both groups, suggests that inflammation is involved also during early remodeling. At 10 days, there was a large proportion of the phagocytes (CD11b^+^) that did not express CD68. We don’t know what these cells are. Furthermore, there was an increase in cytotoxic T cells (CD3^+^ CD8a^+^) over time in the full loading group, which is unexplained.

Previous data using this model show that loading leads to a bigger tendon callus, although not necessarily with better material properties^4^. Because loading in the current study was associated with a prolonged inflammation, it might be suggested that inflammation can increase callus tissue mass while causing delayed improvement of tissue quality. Interestingly, the human Achilles tendon heals primarily without improvement of tissue quality during several months; instead, the tissue mass increases^22^.

In summary, loading delayed the shift from an M1 dominated type of inflammation to a type dominated by M2 macrophages and T_reg_ cells, in the rat Achilles tendon healing model. The clinical implications of this are uncertain.

## Materials and Methods

### Study design

Female Sprague-Dawley rats were used (n = 48; 11–12 weeks old). Loading was reduced in 24 randomly chosen rats by means of Botox injections. This has led to robust reduction in strength of the healing tendon in several previous studies^4^. The Achilles tendon was transected in all rats, and allowed to heal spontaneously without suture. Immune cells from the healing tendons were evaluated by flow cytometry 1, 3, 5 and 10 days after surgery. For some rats, the number of cells was insufficient for a high quality flow cytometry. In those cases, 2 rats were pooled and regarded as a single sample. This produced a sample size of n = 5 for full loading group day 3, n = 5 for Botox group day 3 and day 5, and n = 3 for Botox group day 10. In all other groups the sample size was 6. All experiments were approved by the Regional Ethics Committee for animal experiments in Linköping and adhered to the institutional guidelines for care and treatment of laboratory animals. The rats were housed 2 or 3 per cage and given food and water ad libitum.

### Unloading

For Botox injections, the rats were anesthetized with isoflurane 3 days before tendon transection and the right hind leg was shaved. Botulinum toxin (Botox, Allergan, Irvine, CA) was injected into the gastrocnemius lateralis and medialis and the soleus muscles at a dose of 1 U in each muscle. The total dose was 3 U and 0.06 mL per animal. The rats were allowed free cage activity for 3 days until surgery. All rats were inspected and showed limping due to paralysis before surgery and during the entire experiment.

### Surgery

Rats were anesthetized with isoflurane gas (Forene, Abbot Scandinavia, Solna, Sweden). Antibiotics (25 mg/kg, Oxytetracycline, Engemycin; Intervet, Boxmeer, The Netherlands) were given preoperatively and analgesics (0.045 mg/kg, Buprenorphine, Temgesic; Schering-Plough, Brussels, Belgium) were given subcutaneously pre and postoperatively. The surgery was performed under aseptic conditions. The skin on the right Achilles tendon was shaved and cleaned with chlorhexidine ethanol. A transverse skin incision was made lateral to the Achilles tendon, and the tendon complex was exposed. The plantaris tendon was removed and the Achilles tendon was cut transversely and the tendon was left unsutured to heal spontaneously. Thereafter, the wound was closed by two stitches.

### Tissue harvest and retrieval of single cells

Rats were euthanized by CO_2_ and the tendons dissected out. The former tendon stumps inside the tendon callus were visualized by trans-illumination. The distance between them generally exceeded 5 mm. Two parallel cuts about 4 mm apart were made perpendicular to the direction of the tendon in the middle of the former defect. In this way, old tendon tissue could be excluded from analysis. The excised specimens were placed in digestion buffer (RPMI 1640 with, 5% heat inactivated fetal bovine serum, and 10 mM HEPES). The specimens were minced into small pieces using scissors and then incubated with 1 mg/mL Collagenase D (Roche) and 30 μg/ml DNase (Roche) at 37 °C for 45 min. Cells were separated using a 70 μm cell strainer (Fisher scientific). Cells were washed, and ACK lysis buffer (155 mM NH3Cl, 10 mM KHCO3, and 88 μM EDTA) was used to remove the RBC. In the last step, Trypan blue (Life technologies) was used to count live cells^23^.

### Flow cytometric phenotyping of immune cells

Antibodies were CD45-PE-Cy7, CD3-AF647, CD4-PE, CD25-BV510, Foxp3-AF488, CD8a-PerCP from Biolegend and CD11b-AF700, CD68-BV510 from AbD serotec and CCR7-AF647, CD206-FITC from Bioss and CD163-PE from LSBio. Single cell suspensions were first stained with 50 μl antibody mixture for the surface markers CD45, CD11b, CD163, CD206, CCR7 (macrophage panel), or CD45, CD3, CD4, CD25, CD8 (T cell panel), for 30 minutes in room temperature. Cells were then washed once and incubated with Cytofix/Cytoperm for intracellular staining according to the manufacturer’s instructions (BD Biosciences) at 4 °C for 20 min. Fixed and permeabilized samples were stained with intracellular markers CD68 (for macrophage panel) or Foxp3 (for T cell panel) diluted in Perm/Wash buffer for 30 minutes. Cells were then washed 3 times with Perm/Wash buffer before being fixed in 1% paraformaldehyde. Data were acquired using Gallios Flow Cytometer (Beckman Coulter) and fluorescence minus one (FMO) samples was used to set the gates. Evaluation was performed using FlowJo v10 software.

### Statistics

For testing the effect of loading, we used one single specific hypothesis. This hypothesis was defined (time point and the relevant markers) on the basis of descriptive data from full loading rats, before the Botox data were available. The specific hypothesis (difference in CCR7/CD206 ratio at day 5) was tested with Student’s t-test. After performing that test, a secondary hypothesis was formulated, namely that there is a difference in the ratio T_reg_ cells (CD3^+^CD4^+^CD25^+^Foxp3^+^) out of all T helper cells (CD3^+^CD4^+^) on day 5, which was tested in the same way. For all other markers, we performed post-hoc testing based on t-statistics without correction for multiple testing.

## Additional Information

**How to cite this article**: Blomgran, P. *et al*. A possible link between loading, inflammation and healing: Immune cell populations during tendon healing in the rat. *Sci. Rep.* **6**, 29824; doi: 10.1038/srep29824 (2016).

## Supplementary Material

Supplementary Information

## Figures and Tables

**Table 1:** CCR7/CD206 ratio and T_reg_ cell/T helper cells ratio over time.

Time after tendon transection	CCR7/CD206 (M1/M2)		T_reg_/T helper cells	
	Botox Mean (SD)	Full loading Mean (SD)	Botox Mean (SD)	Full loading Mean (SD)
Day 1	1.95 (0.41)	1.80 (0.42)	0.03 (0.01)	0.06 (0.03)
Day 3	2.21 (0.77)	7.01 (1.53)	0.05 (0.03)	0.05 (0.03)
Day 5	1.80 (0.27)	2.80 (0.41)	0.07 (0.02)	0.03 (0.01)
Day 10		1.37 (0.20)		0.07 (0.04)
