# Supplementary material for: A possible link between loading, inflammation and healing: Immune cell populations during tendon healing in the rat
Source: Sci Rep. 2016 Jul 13;6:29824. doi: 10.1038/srep29824 (PMC4942825; doi:10.1038/srep29824)
Supplement: Supplementary Information [file srep29824-s1.pdf]

Supplementary Information

A possible link between loading, inflammation and healing: Immune cell populations during tendon healing in the rat

Parmis Blomgran[1], Robert Blomgran[2], Jan Ernerudh[3] and Per Aspenberg[1]
[1] Orthopaedics, [2] Division of Medical Microbiology, [3] Division of Clinical Immunology
Department of Clinical and Experimental Medicine, Linkoping University, Sweden

Corresponding Author: parmis.blomgran@gmail.com

“Supplementary Table S1”. Cell surface markers used.

**Marker**	**Cell type**	**Company**
CD45	Pan-leukocyte	Biolegend
CD11b	Phagocyte	AbD serotec
CD68	Pan-macrophage	AbD serotec
CCR7	M1 macrophage	Bioss
CD163	M2 macrophage	LSBio
CD206	M2 macrophage	Bioss
CD3	T cell	Biolegend
CD4	T helper cell	Biolegend
CD25	Regulatory T cell (In combination with Foxp3)	Biolegend
Foxp3	Regulatory T cell	Biolegend
CD8a	Cytotoxic T cell	Biolegend

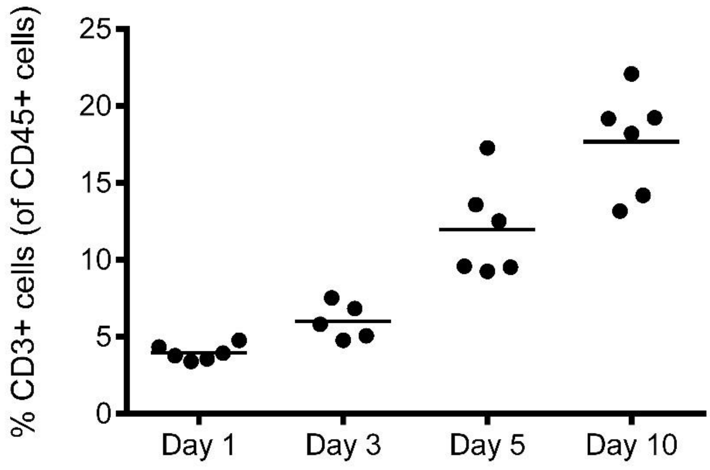

Supplementary Figure S1. The proportion of T cells ($CD3^+$) in the healing tissue presented as percent of all leukocytes ($CD45^+$) over time in the fully loaded group.

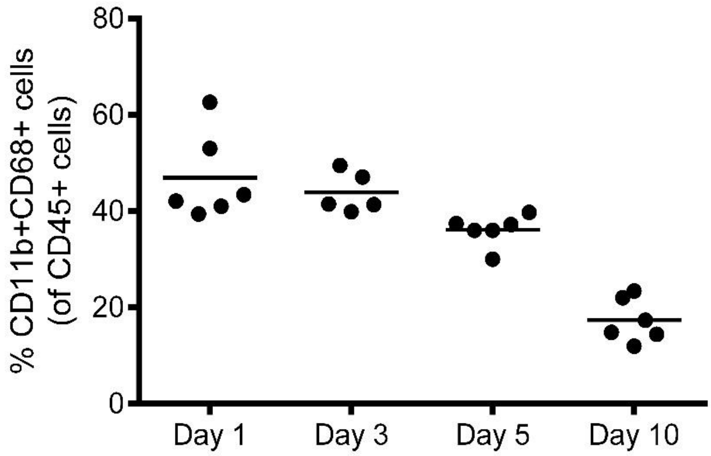

Supplementary Figure S2. The proportion of macrophages ($CD11b^+CD68^+$) in the healing tissue presented as percent of all leukocytes ($CD45^+$) over time in the fully loaded group.

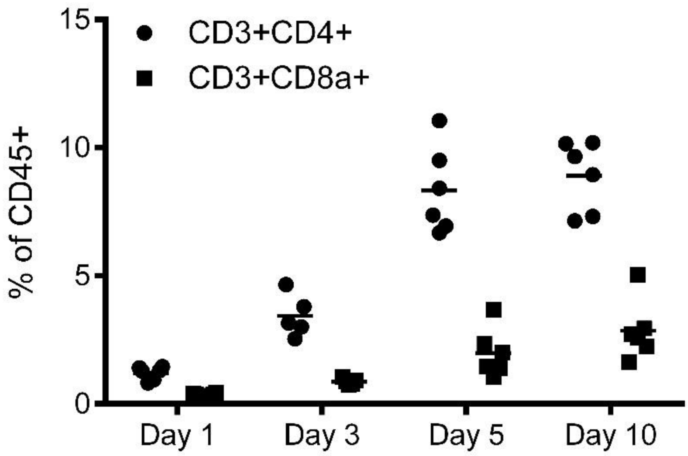

Supplementary Figure S3. The proportion of T helper ($CD3^+CD4^+$) and cytotoxic T cells ($CD3^+CD8a^+$) in the healing tissue presented as percent of all leukocytes ($CD45^+$) over time in the fully loaded group.
